# Comparison of a Novel Computerized Analysis Program and Visual Interpretation of Cardiotocography

**DOI:** 10.1371/journal.pone.0112296

**Published:** 2014-12-01

**Authors:** Chen-Yu Chen, Chun Yu, Chia-Chen Chang, Chii-Wann Lin

**Affiliations:** 1 Institute of Biomedical Engineering and College of Medicine, National Taiwan University, Taipei, Taiwan; 2 Department of Obstetrics and Gynecology, Mackay Memorial Hospital, Taipei, Taiwan; 3 Mackay Medical College, Taipei, Taiwan; 4 Mackay Junior College of Medicine, Nursing, and Management, Taipei, Taiwan; Université de Montréal, Canada

## Abstract

**Objective:**

To compare a novel computerized analysis program with visual cardiotocography (CTG) interpretation results.

**Methods:**

Sixty-two intrapartum CTG tracings with 20- to 30-minute sections were independently interpreted using a novel computerized analysis program, as well as the visual interpretations of eight obstetricians, to evaluate the baseline fetal heart rate (FHR), baseline FHR variability, number of accelerations, number/type of decelerations, uterine contraction (UC) frequency, and the National Institute of Child Health and Human Development (NICHD) 3-Tier FHR classification system.

**Results:**

There was no significant difference in interobserver variation after adding the components of computerized analysis to results from the obstetricians' visual interpretations, with excellent agreement for the baseline FHR (ICC 0.91), the number of accelerations (ICC 0.85), UC frequency (ICC 0.97), and NICHD category I (kappa statistic 0.91); good agreement for baseline variability (kappa statistic 0.68), the numbers of early decelerations (ICC 0.78) and late decelerations (ICC 0.67), category II (kappa statistic 0.78), and overall categories (kappa statistic 0.80); and moderate agreement for the number of variable decelerations (ICC 0.60), and category III (kappa statistic 0.50).

**Conclusions:**

This computerized analysis program is not inferior to visual interpretation, may improve interobserver variations, and could play a vital role in prenatal telemedicine.

## Introduction

Cardiotocography (CTG), also known as electronic fetal monitoring, is a common tool for recording fetal heart rates (FHRs) and uterine contractions (UCs) to evaluate fetal conditions and uterine activities during pregnancy, particularly during the active phase of labor. The accuracy of a CTG diagnosis depends on the analysis of characteristic FHRs and UCs. According to the criteria and consensuses of the National Institute of Child Health and Human Development (NICHD) in April 2008 [1], a complete CTG interpretation includes both qualitative and quantitative descriptions of FHR (i.e., baseline, baseline variability, acceleration, early deceleration, late deceleration, variable deceleration, prolonged deceleration, recurrent deceleration, and sinusoidal pattern) and UC (i.e., baseline uterine tone, contraction frequency, duration, and strength). Conventional visual CTG interpretation is limited, and many previous studies have documented high intraobserver and interobserver variations [2]–[4]. Computerized analysis can preclude these disadvantages, decrease the examination time, and improve clinical care [4], [5]. Recently, several commercial computerized CTG systems have been made available for clinical use [6]–[8]. Most of these instruments could detect baseline FHR, baseline FHR variability, and numbers of accelerations/decelerations, but they rarely differentiated deceleration characteristics. However, it is essential to distinguish the types of decelerations because of the different prognoses that accompany them [9].

In 2008, the NICHD Working Group recommended the 3-Tier classification system to categorize FHR patterns [1]. Category I FHR records are normal and predictive of a normal fetal acid-base status; category II FHR records are indeterminate, and category III FHR records are abnormal. Following the NICHD criteria, we developed an objective and quantitative CTG analysis program using the Laboratory Virtual Instrumentation Engineering Workbench (LabVIEW, National Instrument Inc., USA) graphical software system. Our previous preliminary data have revealed the potential of this computerized program [10]. The intrapartum CTG tracings are more complex and visual interpretation of the intrapartum CTG tracings has shown insufficient reliability in comparison to computerized analysis [11]. Based on the NICHD 3-Tier classification system, the present study was designed to assess the agreement of intrapartum CTG tracings between the results from the computerized CTG analysis program and those from the visual interpretations by eight obstetricians. We hypothesized that this computerized analysis program may improve interobserver variations, and could play a vital role in prenatal telemedicine.

## Methods

**Figure 1:**
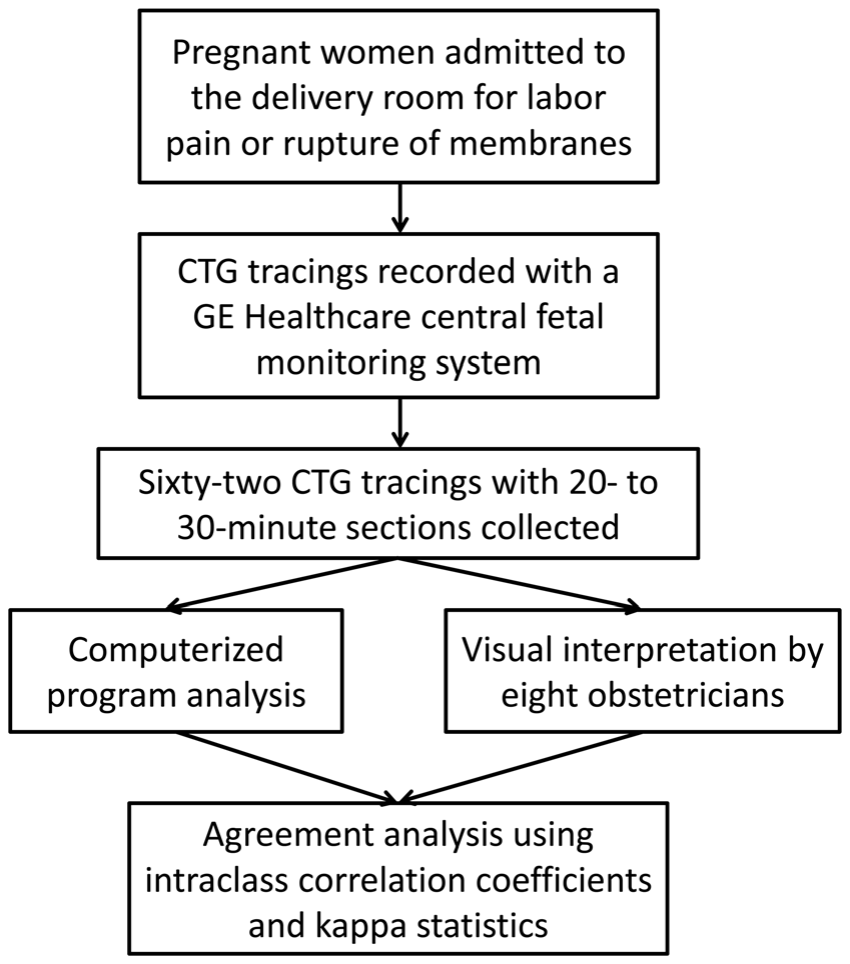
A flow diagram of this study.

We conducted a study to analyze the intrapartum CTG tracings acquired at Mackay Memorial Hospital, a tertiary referral center, between March 2011 and September 2011 (Figure 1). Sixty-two CTG tracings with 20- to 30-minute sections were collected from different pregnant women upon admission to the delivery room for labor pain, with cervical dilation (≧3 cm) or rupture of membranes. The inclusion criteria were as follows: (1) singleton gestation, (2) gestational age ≧37 wks, (3) no known medical problems in the mother, and (4) no known congenital anomalies in the fetus. A continuous-wave Doppler ultrasound transducer was strapped to the pregnant abdomen over the area of detectable fetal heartbeats to monitor the FHRs. A tocodynamometer was strapped to the pregnant abdomen on the fundal area of the uterus to measure the pressure of UCs. The FHR and UC signals were recorded with a GE Healthcare central fetal monitoring system, and the data were uploaded to computers. This study was submitted to the Mackay Memorial Hospital Institutional Review Board, which advised that formal ethical approval was unnecessary, as this study constituted a retrospective audit/service evaluation. The CTG tracings included in this study were obtained from preexisting databases, and the authors had no access to the patients' personal information prior to the anonymization. All personal identifiers were anonymized prior to the computerized CTG analysis and visual interpretation by the obstetricians. Furthermore, the patient demographics were concealed and linked by unique personal identification numbers; therefore, the patients' treatments were unaffected by this study.

Table 1 shows the 2008 NICHD criteria, including the definition of each FHR pattern and the 3-Tier classification system, which was used to guide our analyses. Some FHR patterns required further quantitative measurement in the computer system. First, the definition of absent and minimal baseline variability were clarified. Absent baseline variability was defined as “FHR amplitude range undetectable” in the NICHD. However, FHR variability with 0 beats per minute (bpm) does not exist in the living fetus, and we used the definition of Parer et al., with an amplitude of <2 bpm as absent variability and an amplitude between 2 and 5 bpm as minimal variability [8]. Second, the lag time of late deceleration had to be quantified. No definite lag time duration was described by the NICHD; therefore, we adopted the classification scheme of Caldeyro-Barcia et al., which states that the delay of the FHR nadir occurring after the UC peak should be ≧18 seconds [12]. Third, the amplitude of the FHR sinusoidal pattern had to be clearly defined. The NICHD Working Group defined the sinusoidal pattern as a smooth, sine wave–like undulating pattern, with a cycle frequency of 3 to 5 times per minute that persists for ≧20 minutes [1]. To quantitatively measure the amplitude of the sinusoidal pattern, we used the definition of Modanlou et al., with an amplitude of 5 to 15 bpm [13].

**Table 1:** Characteristics of fetal heart rates and the 3-tier categorization system.

Parameter		Definition	Code
Baseline	Normal	≧110 bpm and ≤160 bpm	1
	Tachycardia	>160 bpm, ≧10 min	2
	Bradycardia	<110 bpm, ≧10 min	3
Baseline variability	Absent	<2 bpm	4
	Minimal	2–5 bpm	5
	Moderate	6–25 bpm	6
	Marked	>25 bpm	7
Acceleration		An increase ≧15 bpm, ≧15 sec and <2 min (≧32 wks)	8
Deceleration	Early	Onset to nadir ≧30 sec	9
		No lag time	
	Late	Onset to nadir ≧30 sec	10
		Lag time (≧18 sec)	
	Variable	Onset to nadir <30 sec	11
		A decrease ≧15 bpm, ≧15 sec and <2 min	
	Prolonged	A decrease ≧15 bpm, ≧2 min and <10 min	12
	Recurrent	Deceleration ≧50% of uterine contractions in a 20-min window	13
Sinusoidal pattern		≧5 bpm and <15 bpm	14
		3–5 cycles/min, ≧20 min	
Category I	1∩6 – (10∪11∪12∪14).		
Category II	3 – (3∩4), 2, 5, 4 – (4∩13), 7, 12, 11∩13∩(5∪6), or 10∩13∩6		
Category III	4∩3, 4∩13∩(10∪11), or 14		

bpm, beats per minute.

The analysis method was developed using LabVIEW 2010 software, which consists of a powerful graphical programming language. LabVIEW software is commonly used to process complicated measurements and automation applications in engineering and science.

**Figure 2:**
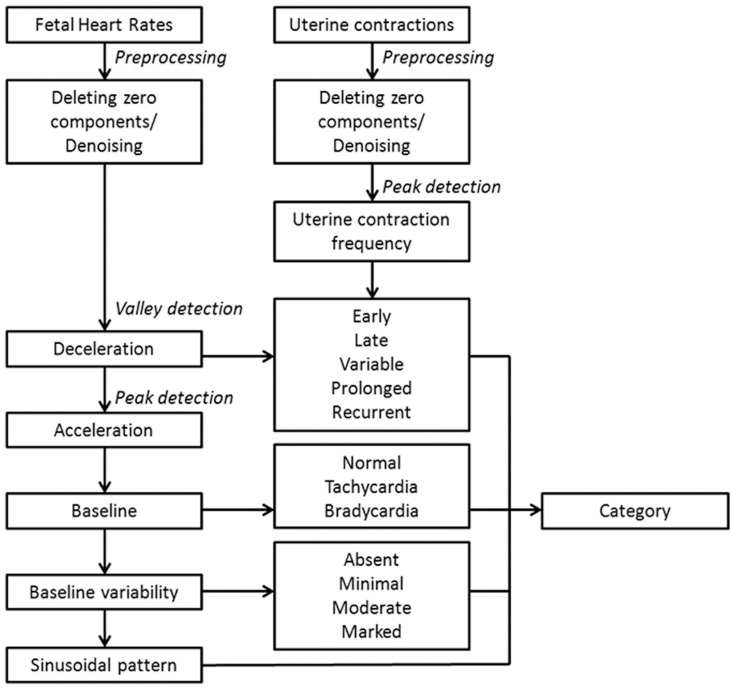
A flow diagram of the software algorithm.

**Figure 3:**
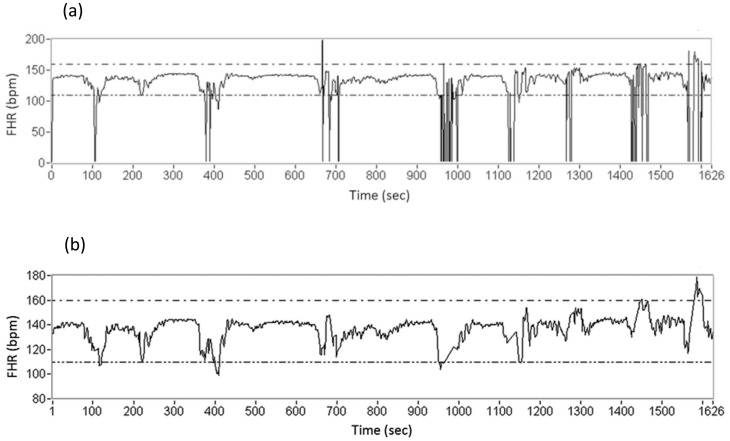
An example of the fetal heart rate tracings: (a) the original pattern; (b) the pattern after deleting the lost signal components, eliminating the noise, and filling in using linear interpolation.

Figure 2 shows the flow diagram of our software algorithm. FHR tracings usually mix with noise and lose some signal components, which can complicate the analysis. The first step of our analysis included deleting the lost signal components and removing noise from the FHR data (Figure 3). After deleting the data regions with no signals, the segments were completed by linear interpolation. A 21-point weighted moving average (MA) filter was utilized to estimate the main tendency of the FHR patterns to reduce the influence of sudden peak/valley values and noise [14]. Similarly, UC tracings were filtered with the MA filter, and the second derivative of the UC amplitude was calculated to determine UC peaks. To increase the accuracy of the algorithm, we used the threshold (mean + standard deviation) to assist in the determination of UC peaks.

We then used the valley detection method to determine the nadirs of FHR decelerations [15]. The valley detection method is based on an algorithm that fits a quadruple polynomial to sequential groups of data points. After employing the valley detection method, we identified the nearest maximum in front of the FHR nadir and calculated the time difference between the maximum and the nadir. The nadir location of FHR deceleration was compared with the UC peak location to classify early, late, variable, and/or recurrent decelerations. The durations of FHR decelerations were calculated to determine the presence of prolonged decelerations. Similarly, we used the peak detection method to determine the FHR accelerations [15].

After excluding the FHR accelerations and decelerations, we calculated the mean FHR as baseline and the standard deviation as baseline variability during every 10-minute window. The determination of FHR baseline in the CTG tracings was presented using the average value of each time period, and variability was presented using the highest values of each time period. Tachycardia and bradycardia were also determined. Finally, the distributions of peak-to-peak and trough-to-trough intervals in the FHR tracings were checked to exclude the possibility of sinusoidal patterns. Categorization was determined after all parameters were calculated. If a category II or III FHR was observed, the categorization would not be established until the full CTG tracing was performed. Thus, a category II or III FHR was not diagnosed if there was an improvement of FHR within the observation period, and there were no overlapped diagnoses between categories I, II and III in each CTG record. The original CTG tracings and results of their analyses were stored in a MySQL database, which allowed the clinicians to review the data using Microsoft Access and Excel.

CTG tracings were examined by the first author (Chen CY, an attending doctor with seventeen years of clinical prenatal care experience) to ensure there were adequate records that included representative data from categories I, II, and III. The CTG records were analyzed using our LabVIEW program and individually interpreted by eight obstetricians (obstetrician A to H) with clinical prenatal care experience who were in practice between three and six years. All obstetricians provided independent diagnoses of the CTG records and were unaware of the results from the other examiners or computerized analysis. With regard to the baseline FHR, baseline variability, number of accelerations, number/type of decelerations, UC frequency, and categories, the interobserver variations among the eight obstetricians were analyzed. Furthermore, agreements between the computerized CTG analysis and visual interpretation were also compared.

SPSS version 18.0 (SPSS Inc., Chicago, IL, USA) was used for the statistical analysis. Intraclass correlation coefficients (ICCs) for the continuous variables and kappa statistics for the categorical variables were used to evaluate the interobserver variations in the visual interpretations and the agreements between the computerized CTG analyses and visual interpretations. Interobserver variability was interpreted as poor (<0.21), fair (0.21–0.40), moderate (0.41–0.60), good (0.61–0.80), and excellent (0.81–1.00) agreement based on the respective ICC or kappa values [16], [17].

## Results

Sixty-two intrapartum CTG tracings with 20- to 30-minute sections were collected and independently analyzed using the computerized analysis program and eight obstetricians. Table 2 shows the characteristics of the CTG tracings. The mean gestational age was 38 wks (range 37 to 40 wks). All eight obstetricians identified a mean of 142 accelerations, 14 early decelerations, 48 late decelerations, 74 variable decelerations, and 450 UCs. The computerized analysis program identified 142 accelerations, 17 early decelerations, 55 late decelerations, 70 variable decelerations, and 443 UCs. Category III was diagnosed six times by obstetrician H; five times by obstetrician A; four times by the computerized analysis and obstetricians C, D, and F; three times by obstetrician E; and twice by obstetricians B and G. Among 62 CTG tracings analyzed by the analysis program and eight obstetricians, there were 558 CTG records. In total, 198 (35.5%) CTG tracings were classified as category I, while 326 (58.4%) were classified as category II, and 34 (6.1%) were classified as category III.

**Table 2:** Characteristics of 62 cardiotocography tracings obtained by computerized analysis and visual interpretations by eight obstetricians.

	Computer	A	B	C	D	E	F	G	H
Mean baseline FHR (bpm)	147	147	146	146	148	146	147	147	148
Baseline variability									
Absent	6	7	3	5	5	5	5	2	7
Minimal	16	16	18	14	18	16	17	22	19
Moderate	40	39	40	43	39	41	40	38	36
Marked	0	0	1	0	0	0	0	0	0
No. of accelerations	142	133	167	119	129	110	147	164	169
No. of early decelerations	17	13	19	17	12	6	14	8	20
No. of late decelerations	55	58	28	33	69	34	49	46	69
No. of variable decelerations	70	53	83	76	57	76	69	98	81
No. of prolonged decelerations	7	5	7	8	5	6	4	8	6
No. of recurrent decelerations	18	18	14	18	20	17	18	20	22
No. of UCs	443	448	430	455	453	428	458	467	463
Category									
I	21	21	22	24	23	23	22	21	21
II	37	36	38	34	35	36	36	39	35
III	4	5	2	4	4	3	4	2	6

FHR, fetal heart rate; bpm, beats per minute; UCs, uterine contractions.

Interobserver variations in the visual interpretations of the data and results of the computerized analysis are shown in Table 3. Agreement among the visual interpretation data was excellent for the baseline FHR (ICC 0.91), number of accelerations (ICC 0.84), presentation of prolonged decelerations (kappa statistic 0.82), presentation of recurrent decelerations (kappa statistic 0.82), UC frequency (ICC 0.97), and category I (kappa statistic 0.90). Agreement was good for the baseline variability (kappa statistic 0.67), number of early decelerations (ICC 0.78), number of late decelerations (ICC 0.65), category II (kappa statistic 0.78), and overall categories (kappa statistic 0.80). Agreement was moderate for the number of variable decelerations (ICC 0.59) and category III (kappa statistic 0.48).

**Table 3:** Interobserver variations between the results of the eight obstetricians' visual interpretations and the computerized analysis.

	Visual interpretation only				Visual interpretation and computerized analysis			
	ICC	Kappa statistic	95% CI	Agreement	ICC	Kappa statistic	95% CI	Agreement
Baseline FHR	0.91		0.88–0.94	Excellent	0.91		0.88–0.94	Excellent
Baseline variability		0.67	0.51–0.83	Good		0.68	0.51–0.84	Good
Acceleration	0.84		0.79–0.89	Excellent	0.85		0.80–0.90	Excellent
Early deceleration	0.78		0.71–0.84	Good	0.78		0.71–0.84	Good
Late deceleration	0.65		0.56–0.74	Good	0.67		0.59–0.76	Good
Variable deceleration	0.59		0.50–0.69	Moderate	0.60		0.51–0.70	Moderate
Prolonged deceleration		0.82	0.58–1.00	Excellent		0.82	0.58–1.00	Excellent
Recurrent deceleration		0.82	0.66–0.97	Excellent		0.82	0.67–0.97	Excellent
UC frequency	0.97		0.96–0.98	Excellent	0.97		0.96–0.98	Excellent
Category								
I		0.90	0.81–1.00	Excellent		0.91	0.81–1.00	Excellent
II		0.78	0.62–0.93	Good		0.78	0.63–0.93	Good
III		0.48	0.15–0.80	Moderate		0.50	0.17–0.83	Moderate
Overall		0.80	0.66–0.93	Good		0.80	0.67–0.94	Good

FHR, fetal heart rate; UC, uterine contraction; ICC, intraclass correlation coefficient; CI, confidence interval.

After adding the components of the computerized CTG analysis to the obstetricians' visual interpretations, the ICC and kappa statistic values were not obviously affected (Table 3). Agreement was still excellent for the baseline FHR (ICC 0.91), number of accelerations (ICC 0.85), presentation of prolonged decelerations (kappa statistic 0.82), presentation of recurrent decelerations (kappa statistic 0.82), UC frequency (ICC 0.97), and category I (kappa statistic 0.91). Agreement was good for the baseline variability (kappa statistic 0.68), number of early decelerations (ICC 0.78), number of late decelerations (ICC 0.67), category II (kappa statistic 0.78), and overall categories (kappa statistic 0.80). Agreement was moderate for the number of variable decelerations (ICC 0.60) and category III (kappa statistic 0.50).

The agreements between the computerized CTG analysis and visual interpretations of the eight individual obstetricians are shown in Table 4. The computerized analysis showed excellent agreement with the eight individual obstetricians for NICHD category I (kappa statistics 0.82 to 0.93), good agreement for category II (kappa statistics 0.70 to 0.87) and overall categories (kappa statistics 0.72 to 0.88), but inconsistent agreement for category III (kappa statistics 0.10 to 0.78). We further compared category III FHR tracings between computerized analysis and visual interpretation (Table 5). No FHR tracings of category III classified by visual interpretation were classified as category I by computerized analysis.

**Table 4:** Intraclass correlation coefficients or kappa statistics (*) between the results from the computerized analysis and the visual interpretations of eight individual obstetricians.

	A	B	C	D	E	F	G	H
Baseline FHR	0.98	0.81	0.94	0.92	0.95	0.94	0.94	0.94
Baseline variability	0.72*	0.68*	0.70*	0.78*	0.71*	0.74*	0.59*	0.64*
Acceleration	0.92	0.85	0.82	0.93	0.88	0.85	0.89	0.89
Early deceleration	0.87	0.83	0.84	0.83	0.59	0.78	0.59	0.82
Late deceleration	0.89	0.60	0.60	0.85	0.74	0.82	0.70	0.84
Variable deceleration	0.67	0.74	0.62	0.67	0.45	0.88	0.71	0.46
Prolonged deceleration	0.82*	0.84*	0.92*	0.63*	0.74*	0.70*	0.92*	0.74*
Recurrent deceleration	0.84*	0.75*	0.84*	0.77*	0.88*	0.84*	0.70*	0.71*
UC frequency	0.98	0.96	0.98	0.97	0.95	0.97	0.97	0.98
Category								
I	0.93*	0.82*	0.83*	0.93*	0.86*	0.89*	0.93*	0.93*
II	0.83*	0.70*	0.70*	0.87*	0.77*	0.77*	0.73*	0.87*
III	0.64*	0.30*	0.47*	0.73*	0.55*	0.47*	0.10*	0.78*
Overall	0.85*	0.72*	0.73*	0.88*	0.79*	0.79*	0.75*	0.88*

FHR, fetal heart rate; UC, uterine contraction.

**Table 5:** Comparison of category III between computerized analysis and visual interpretation.

	Computer	A	B	C	D	E	F	G	H
No. of category III	4	5	2	4	4	3	4	2	6
No. of category III overlapping with computer		3	1	2	3	2	2	0	4
No. of category II by computer		2	1	2	1	1	2	2	2
No. of category I by computer		0	0	0	0	0	0	0	0

## Discussion

The outcome of this computerized analysis was similar to that of the visual interpretation. After adding the results of the computerized CTG analysis to the obstetricians' visual interpretations, all agreements were not obviously affected. According to comfort, signal quality and fulfillment of the criteria of category I, 20- to 30-minute sections of intrapartum CTG tracings were recorded. Initially, obtaining a reproducible measurement of baseline FHR is important for objective CTG interpretation. Our analysis program yielded excellent intrapartum baseline FHR agreement (ICC 0.91) similar to that obtained using other computer systems (ICC 0.85 to 0.95) [18]–[20]. Moderate FHR variability is a reliable indicator of fetal wellbeing in the absence of fetal metabolic academia [1]. The reliability of baseline variability (kappa statistic 0.68) in our analysis program was greater than that observed in previous studies (kappa statistics, 0.15 to 0.38) [18], [21]–[23]. Excellent agreement of our analysis program was also found in the number of accelerations (ICC 0.85), and the validity of this parameter was similar to the study by Di Lieto et al. (ICC 0.87) and better than the results of other previous reports [18], [22]–[25].

The deceleration agreement in previous studies was not better than that in the present study, with the exception of one study by Taylor et al., which yielded an ICC value of 0.93 [18]. In the visual interpretation results of our study, agreement was moderate for the number of variable decelerations (ICC 0.59). Unsurprisingly, there was also moderate agreement for the number of variable decelerations after the computerized analysis was added to the obstetricians' visual interpretations (ICC 0.60). NICHD category III includes absent FHR variability combined with recurrent variable decelerations; thus, moderate agreement for category III was noted in our study (kappa statistic 0.50). In the Blackwell et al. study, agreement for category III was poor mainly because of the lack of agreement between absent and minimal variability [26]. To the best of our knowledge, no previous studies have evaluated the agreement between computerized CTG and visual interpretations for the 3-Tier classification system.

CTG is still the most prevalent and acceptable instrument used by obstetricians to detect fetal conditions, despite the fact that many other ancillary methods, such as fetal scalp pulse oximetry, fetal scalp blood pH or lactate measurement, and fetal electrocardiogram (ECG) ST-segment analysis (STAN, Neoventa Medical, Gothenburg, Sweden), are also available [27]–[29]. These methods are usually practical after membrane rupture and are more invasive; furthermore, no conclusive evidence regarding fetal scalp pulse oximetry and improvement of prenatal outcomes is available [30], [31]. Recently, a transabdominal fetal ECG recording method (AN24, Monica Healthcare, Nottingham, UK) was developed and has been reported to correlate well with scalp electrode recordings [32]. However, ECG recordings have typically been used together with CTG recordings to predict fetal conditions [29].

**Figure 4:**
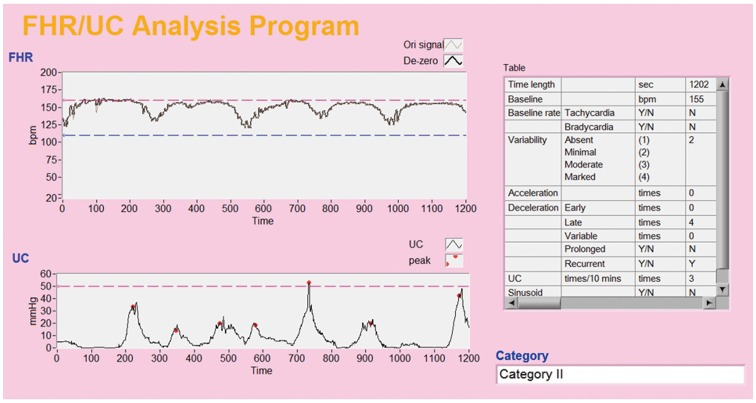
A user friendly interface from the LabVIEW software program that is used for prenatal telemedicine.

Poor reproducibility in the visual interpretation of CTG records diminishes the clinical value of CTG and may increase the rates of cesarean section and instrumental vaginal delivery (i.e., using forceps or vacuum extraction) [2], [3], [33]. Several commercial computerized CTG systems have been introduced for clinical use and have attempted to improve the limitations of conventional visual interpretation [7]–[9]. Furthermore, previous studies have developed various mathematical algorithms for the computerized analysis of CTG tracings, including those from the time domain to the frequency domain, from linear to nonlinear analysis, and from expert systems to neural networks [11], [34]–[37]. It is difficult to assess which of these methods is best. Most studies have evaluated the baseline FHR, baseline variability, and even numbers of accelerations and decelerations, but few have clearly differentiated among the type of decelerations, particularly in the frequency domain. Chung et al. used the Turbo Pascal programming language to analyze intrapartum CTG tracings based on the raw fetal ECG data obtained from fetal scalp clips [11]. Differing from the definitions proposed by the NICHD [1], the authors defined early decelerations as FHR minima occurring within 20 seconds of UC maxima, late decelerations as FHR minima occurring 20 to 60 seconds after UC maxima, and variable decelerations as FHR minima occurring more than 20 seconds prior to or 60 seconds after UC maxima. Compared with umbilical arterial blood pH and base excess at delivery, their software program could predict a pH value of <7.15 with an accuracy of 77% and a base excess of <−8 mmol/l with an accuracy of 81%. In contrast to textual programming languages, such as Turbo Pascal, LabVIEW is a graphical programming language with a user-friendly interface that is useful not only for clinicians but also for pregnant women (Figure 4). An expert system to support clinical decision-making was recently developed (K2 Medical Systems, Plymouth), and the software is able to evaluate the baseline FHR, baseline variability, accelerations, type and timing of decelerations, and UC pattern. The inventors are completing a prospective, multicenter randomized controlled trial, and the final results are expected in the future.

The purpose of our study was designed to assess agreement between computerized analysis and visual interpretation, not accuracy; thus, this study has some limitations. First, no universal umbilical artery blood gases were evaluated at birth; therefore, we could not analyze the relationship between the FHR patterns and neonatal outcomes. Second, no intraobserver variations were evaluated in the obstetricians' visual interpretations. Third, we did not ask the mothers to count fetal movements during the intrapartum period, and, therefore, missing information on fetal movement is a setback to this study. Fourth, sinusoidal patterns are frequently observed in cases of fetomaternal Rh incompatibility, which is uncommon in Asians. In this study we did not recruit CTG tracings with sinusoidal FHR patterns, and thus we could not investigate the interobserver variation in these patterns. Furthermore, moderate agreement for category III was noted in this study, either interobserver variations in the obstetricians' visual interpretations or after adding the components of the computerized CTG analysis to the visual interpretations. Therefore, the potential risk of pathological condition still cannot be overlooked when we use this computerized analysis program.

CTG has existed since the 1960s, and some of the definitions have been updated during this development period. For example, it is not meaningful to distinguish between short- and long-term variability at this time because these parameters are visually determined as a unit in clinical practice [1]. From this viewpoint, some research concerning short-term variability might now be of little value. Our analysis program is based on the updated definitions of the NICHD, and it is easy to revise our program because the LabVIEW software provides a block diagram format in which functional icons can be easily added or removed.

Computerized CTG analysis has been investigated for three decades as an alternative to improve the poor reproducibility of visual interpretation of CTG tracings. The NICHD Working Group reasserted that the definitions of electronic fetal monitoring apply not only to visual interpretation but must also be suitable for computerized applications [1]. In this study, we developed a LabVIEW-based CTG analysis software program and verified the validity of this program. This computerized analysis program is not inferior to visual interpretation, may improve interobserver variations, and could play a vital role in prenatal telemedicine. The software also has great potential for integration with commercial instruments made by different manufacturers. Further research will involve the use of this CTG analysis system in prenatal telemedicine.
